# Limitations of a Short Demographic Questionnaire for Bedside Estimation of Patients’ Global Cognitive Functioning in Epilepsy Patients

**DOI:** 10.3389/fneur.2018.00085

**Published:** 2018-03-01

**Authors:** Iris Gorny, Kristina Krause, Anita Albert, Sabrina Schneider, Leona Möller, Lena Habermehl, Adam Strzelczyk, Felix Rosenow, Anke Hermsen, Susanne Knake, Katja Menzler

**Affiliations:** ^1^Epilepsy Center Hessen, Philipps-University, Marburg, Germany; ^2^Epilepsy Center Frankfurt Rhine-Main, Goethe University, Frankfurt, Germany

**Keywords:** epilepsy, Wechsler Adult Intelligence Scale-III, short form, intelligence, cognitive function

## Abstract

**Objectives:**

The German socio-demographic estimation scale was developed by Jahn et al. (1) to quickly predict premorbid global cognitive functioning in patients. So far, it has been validated in healthy adults and has shown a good correlation with the full and verbal IQ of the Wechsler Adult Intelligence Scale (WAIS) in this group. However, there are no data regarding its use as a bedside test in epilepsy patients.

**Methods:**

Forty native German speaking adult patients with refractory epilepsy were included. They completed a neuropsychological assessment, including a nine scale short form of the German version of the WAIS-III and the German socio-demographic estimation scale by Jahn et al. (1) during their presurgical diagnostic stay in our center. We calculated means, correlations, and the rate of concordance (range ±5 and ±7.5 IQ score points) between these two measures for the whole group, and a subsample of 19 patients with a global cognitive functioning level within 1 SD of the mean (IQ score range 85–115) and who had completed their formal education before epilepsy onset.

**Results:**

The German demographic estimation scale by Jahn et al. (1) showed a significant mean overestimation of the global cognitive functioning level of eight points in the epilepsy patient sample compared with the short form WAIS-III score. The accuracy within a range of ±5 or ±7.5 IQ score points for each patient was similar to that of the healthy controls reported by Jahn et al. (1) in our subsample, but not in our whole sample.

**Conclusion:**

Our results show that the socio-demographic scale by Jahn et al. (1) is not sufficiently reliable as an estimation tool of global cognitive functioning in epilepsy patients. It can be used to estimate global cognitive functioning in a subset of patients with a normal global cognitive functioning level who have completed their formal education before epilepsy onset, but it does not reliably predict global cognitive functioning in epilepsy patients in general, who often do not fulfill these criteria. It is therefore not a useful tool to be applied in the general neuropsychological presurgical evaluation of epilepsy patients.

## Introduction

The precise assessment of individual cognitive resources and deficits is important for the comprehensive care of epilepsy patients, especially when epilepsy surgery is a feasible therapeutic approach. For the diagnosis and quantification of cognitive impairment in the individual, test results are compared not only to the specific test norms but also to the level of global cognitive functioning (2). The most commonly used measure of global cognitive functioning is the Wechsler Adult Intelligence Scale (WAIS) (3). However, especially in cognitively impaired patients, administration of the WAIS can be time-consuming, so various short forms have been developed. One common approach is to select a different number of subscales (from 2 to 10) of the WAIS (4, 5). The classifications have been shown to be quite robust and correlate highly with the WAIS, especially when seven or more scales are used (6). A different and even less time consuming approach is to estimate the level of global cognitive functioning by means of socio-demographic variables, such as educational attainment and occupational status (7). Studies with healthy controls showed good estimation rates for individuals whose global cognitive functioning level is within 1 SD of the mean (mean IQ range 85–115) (8). These scales may be useful to estimate premorbid cognitive functioning in patients (9, 10). However, these socio-demographic instruments are subject to cultural limitations and may only be applicable in the country in which they have been developed. In 2013, Jahn et al. published a first social formula for Germany, which showed robust results for healthy controls (1). However, the usefulness of this scale has not been investigated in patients with epilepsy.

## Materials and Methods

### Participants

The study included forty adult patients with medically refractory epilepsy who completed a comprehensive neuropsychological assessment, including a nine scale short form of the German version of the WAIS-III (11) and the German socio-demographic estimation scale by Jahn et al. (1), during their routine presurgical diagnostic process.

As the socio-demographic estimation scale tends to represent the premorbid global cognitive functioning within the normal range and uses mainly educational items, we examined a subsample of 19 patients, who fulfilled the following two criteria: first, global cognitive functioning scores of the WAIS-III short form were within 1 SD of the distribution (IQ score = 85 ≤ *X* ≥ 115) and, second, the onset of epilepsy occurred after completion of the patients’ formal education (including academic and vocational training). Please see Table 1 for demographic and seizure characteristics.

**Table 1:** Demographic and epilepsy characteristics.

	Whole sample*n* = 40Mean(SD)/%/[range]	Sub sample (IQ 85–115)*n* = 19Mean(SD)/%/[range]

Age	35 (11.76) [17–65]	38.79 (10.87) [23–65]
Gender: female	47.5%	36.8%
Years of education	10.8 (1.7)	10.9 (1.6)
Duration of epilepsy (years)	10.37 (8.36)	6.47 (6.87)
Age at seizure onset	25.08 (13.98)	32.37 (11.21)

**Type of epilepsy**		
Temporal lobe epilepsy left/right	35%/30%	42.1%/31.6%
Frontal lobe epilepsy	15%	10.5%
Parieto-occipital	7.5%	–
Multifocal	2.5%	–
Generalized	5%	5.3%
Unknown	5%	10.5%
Seizure frequency per month	7.8 (18.75)	3.97 (7.21)

**Seizure type**		
Focal, not bilateral tonic–clonic	47.5%	36.8%
Focal to bilateral tonic–clonic	47.5%	57.9%
Generalized	5%	5.3%

**Number of antiepileptic drug (AED)**		
1	40%	52.6%
2	52.5%	47.4%
3	7.5%	–
AED (number of patients taking the AED)	Carbamazepin (6), lacosamid (9), lamotrigin (15), levetiracetam (17), oxcarbazepin (5), perampanel (1), topiramat (4), valproat (5), zonisamid (2)	Carbamazepin (3), lacosamid (3), lamotrigin (6), levetiracetam (9), oxcarbazepin (2), topiramat (1), valproat (3)

### Measures

The measures evaluated for this study were the total IQ score of the eleven item socio-demographic estimation scale by Jahn et al. (1) (see also Supplementary Material: gender, birth order, highest level of secondary education, grade point average, highest vocational title, private Internet use, preferred newspapers/magazines, preferred type of literature, population of city of residence, duration of formal instruction in a musical instrument) and the total score of the nine scale short form of the German version of the WAIS-III (vocabulary, similarities, arithmetic, digit span, picture completion, block design, matrix reasoning, digit symbol coding, symbol search).

### Analysis

Statistical analyses were calculated using SPSS (IBM^®^ SPSS^®^ Statistics Version 23). The mean scores of the WAIS-III short form and the socio-demographic estimation scale were compared using dependent sample *t*-tests. Pearson correlations were determined and used as an overall measure of agreement between the two instruments. We also analyzed the relation between the difference of the two instruments and the epilepsy duration using Pearson correlations for the whole group and the subsample. Percent agreement was defined as the percentage of participants with ±5 and ±7.5 IQ score points between the WAIS-III short form and the estimation scale. The five-point criterion represented a stricter criterion and was used by, for example, Jahn et al. (1), and Spinks et al. (8), while the 7.5 criterion was applied by Jahn et al. as a more liberal criterion due to the reliability of the WAIS.

## Results

**Figure 1:**
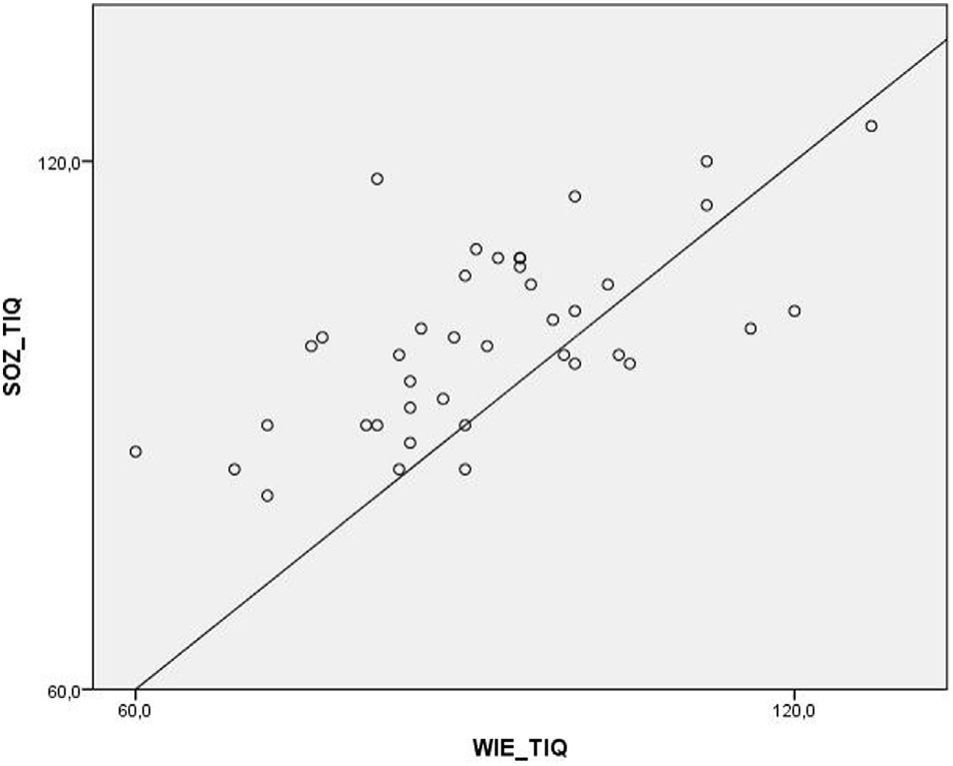
Distribution of the total scores of the WAIS-IV and the socio-demographic estimation scale.

The mean socio-demographic estimation score was significantly higher than the mean WAIS-III short form score (Figure 1).

Both global cognitive functioning scores correlated significantly. The Pearson correlation was *r* = 0.64 (*p* < 0.001) in the whole group. The correlation was slightly lower, but still significant in the subsample (*r* = 0.46, *p* < 0.05).

There was a low correlation between the epilepsy duration (*r* = 0.33, *p* < 0.05) and the difference between the two measures in the whole sample and no significant correlation (*r* = −0.20, *p* = 0.39) in the subsample.

When applying the stricter criterion of an interval of ±5 IQ score points difference, agreement between the two measures was 30% in the whole sample and 42% in the subsample (IQ 85–115). When applying the more lenient criterion of a 7.5 IQ score point difference, agreement rose to 37.5% in the whole sample and 52.6% in the subsample (Table 2).

**Table 2:** Means and classification accuracy of the WAIS-IV and the socio-demographic estimation scale.

	Wechsler Adult Intelligence Scale (WAIS)-III M (SD)	Social scaleM (SD)	*P*	Mean difference	Within 5 points WAIS (%)	Within 7.5 points WAIS (%)
*n* = 40	92.0 (14.0)	100.13 (10.4)	<0.001	8.13	30	37.5
*n* = 19	96.21 (7.67)	101.79 (8.32)	<0.001	5.58	42.1	52.6

## Discussion

This study retrospectively assessed the usefulness of the socio-demographic estimation scale by Jahn et al. (1) for the estimation of global cognitive functioning as measured by a short form of the German version of the WAIS-III in the evaluation of epilepsy patients. While previous reports found no significant differences between the two scores in samples of healthy participants (1, 12), means of the demographic score significantly overestimated the WAIS-III scores in our epilepsy patient sample. The strength of the relationship between these two measures was moderate. These results are in line with the findings of previous studies in other patient populations (9). This overestimation might represent the difference between the premorbid level of global cognitive functioning as estimated by the socio-demographic estimation score and the current level at the time of assessment and might be due to the disorder itself or antiepileptic medication (13). However, classification accuracy in the subsample who had completed their formal education before epilepsy onset was similar to the 36.6% (±5 points) and 51.8% (±7.5 points) reported in healthy subjects (1). The shorter duration of epilepsy in these patients might contribute to this finding. This hypothesis is further supported by a weak correlation between epilepsy duration and discrepancy between the estimation scale and the WAIS-III in the whole group. The higher discrepancy between the two test results in patients with a longer duration of epilepsy might, however, also be influenced by the fact that patients with an early onset of epilepsy more often have severe epilepsy syndromes and an IQ below normal range. Accordingly, we did not find a significant correlation between epilepsy duration and discrepancy between the estimation scale and the WAIS-III in our subsample with an IQ within 1 SD of the mean. This finding and the lower estimation accuracy in the overall sample, which is in line with earlier studies (14), demonstrate the limitations of the estimation scale, which requires a minimum IQ score of 80 and is mainly based on the education level. The usefulness of the socio-demographic estimation scale is therefore limited in epilepsy patients in general due to the high proportion of patients who do not fulfill these criteria (15).

Future studies should ideally include a larger patient sample with different subsamples and make use of the full version of the WAIS instead of a nine scale short form. Since Jahn et al. (1) originally developed their scale in reference to WAIS-II, but provided proxy scores to the WAIS-III, future studies should reflect the recent developments and test revisions and thus employ the German version of the WAIS-IV.

## Conclusion

The socio-demographic scale developed by Jahn et al. (1) overestimates global cognitive functioning in patients with epilepsy and only shows similar accuracy to results in healthy subjects in a subgroup of patients with normal global cognitive functioning up to 1 SD below the mean and who have finished their formal education before epilepsy onset. Therefore, the use of the estimation scale is limited in the population of epilepsy patients. Especially in the context of neuropsychological presurgical evaluations a detailed evaluation of the patient’s level of global cognitive functioning is highly recommended to ensure optimal presurgical evaluation results.

## Ethics Statement

This retrospective study included clinically acquired data. Data were extracted from patient files of patients who had completed a complete pre-surgical neuropsychological evaluation. They were analyzed retrospectively in accordance with patient confidentiality guidelines.

## Author Contributions

IG: acquisition, analysis, interpretation of the data; drafting the work, final approval, and agreement to the work. KK: acquisition, interpretation of the data; revising the work, final approval, and agreement to the work. AA and SS: acquisition of the data; revising the work, final approval, and agreement to the work. LM, AS, and FR: conception of the work; revising the work, final approval, and agreement to the work. AH: conception of the work; acquisition of the data; revising the work, final approval, and agreement to the work. SK and KM: conception and design of the work; revising the work, final approval, and agreement to the work.

## Conflict of Interest Statement

The authors declare that the research was conducted in the absence of any commercial or financial relationships that could be construed as a potential conflict of interest.
