# Supplementary material for: Limitations of a Short Demographic Questionnaire for Bedside Estimation of Patients’ Global Cognitive Functioning in Epilepsy Patients
Source: Front Neurol. 2018 Mar 1;9:85. doi: 10.3389/fneur.2018.00085 (PMC5838021; doi:10.3389/fneur.2018.00085)
Supplement: Supplementary file 1 [file data_sheet_1.pdf]

German socio-demographic estimation scale (Jahn et al, 2013)

Age					
Gender	☐ female	☐ male			
Birth order	☐ no siblings	☐ firstborn	☐ older siblings		
Highest level of high school diploma	☐ Hauptschule (lowest level of high school diploma)	☐ Realschule (medium level of high school diploma)	☐ Abitur (highest level of high school diploma)		
grade point average	☐ 1.0 to 1.9	☐ 2.0 and above			
Typical grade: mathematics	☐ 1.0 to 1.9	☐ 2.0 to 2.9	☐ 3.0 and above		
highest vocational title	☐ without vocational training	☐ vocational title	☐ vocational title with additional qualification	☐ academic (incl. university students)	☐ executive position
Private internet use	☐ no	☐ yes			
preferred newspapers/ magazines	☐ sensational press only	☐ none or regional newspapers/ magazines	☐ national newspapers		
preferred type of literature	☐ none	☐ light fiction	☐ literature, non-fiction,	☐ contemporary literature, litterae humaniores, lyric poetry, specialist books	
population of city of residence	☐ ≤ 20.000	☐ > 20.000			
duration of formal instruction in a musical instrument	☐ never or <5 years	☐ 5 years or longer, during lifetime			
